# A Two-Way Communication between Microglial Cells and Angiogenic Sprouts Regulates Angiogenesis in Aortic Ring Cultures

**DOI:** 10.1371/journal.pone.0015846

**Published:** 2011-01-10

**Authors:** Simin F. Rymo, Holger Gerhardt, Fredrik Wolfhagen Sand, Richard Lang, Anne Uv, Christer Betsholtz

**Affiliations:** 1 Institute of Biomedicine, University of Gothenburg, Gothenburg, Sweden; 2 Vascular Biology Laboratory, London Research Institute - Cancer Research UK, London, United Kingdom; 3 Stem Cell Center, Lund University, Biomedical Center (BMC), Lund, Sweden; 4 The Visual Systems Group, Divisions of Pediatric Ophtalmology and Developmental Biology, Department of Ophthalmology, Cincinnati Children's Hospital Medical Center, University of Cincinnati, Cincinnati, Ohio, United States of America; 5 Division of Vascular Biology, Department of Medical Biochemistry and Biophysics, Karolinska Institutet, Stockholm, Sweden; CRT Dresden, Germany

## Abstract

**Background:**

Myeloid cells have been associated with physiological and pathological angiogenesis, but their exact functions in these processes remain poorly defined. Monocyte-derived tissue macrophages of the CNS, or microglial cells, invade the mammalian retina before it becomes vascularized. Recent studies correlate the presence of microglia in the developing CNS with vascular network formation, but it is not clear whether the effect is directly caused by microglia and their contact with the endothelium.

**Methodology/Principal Findings:**

We combined *in vivo* studies of the developing mouse retina with *in vitro* studies using the aortic ring model to address the role of microglia in developmental angiogenesis. Our *in vivo* analyses are consistent with previous findings that microglia are present at sites of endothelial tip-cell anastomosis, and genetic ablation of microglia caused a sparser vascular network associated with reduced number of filopodia-bearing sprouts. Addition of microglia in the aortic ring model was sufficient to stimulate vessel sprouting. The effect was independent of physical contact between microglia and endothelial cells, and could be partly mimicked using microglial cell-conditioned medium. Addition of VEGF-A promoted angiogenic sprouts of different morphology in comparison with the microglial cells, and inhibition of VEGF-A did not affect the microglia-induced angiogenic response, arguing that the proangiogenic factor(s) released by microglia is distinct from VEGF-A. Finally, microglia exhibited oriented migration towards the vessels in the aortic ring cultures.

**Conclusions/Significance:**

Microglia stimulate vessel sprouting in the aortic ring cultures via a soluble microglial-derived product(s), rather than direct contact with endothelial cells. The observed migration of microglia towards the growing sprouts suggests that their position near endothelial tip-cells could result from attractive cues secreted by the vessels. Our data reveals a two-way communication between microglia and vessels that depends on soluble factors and should extend the understanding of how microglia promote vascular network formation.

## Introduction

Angiogenesis is the process whereby new blood vessels form from preexisting ones by sprouting, splitting, growth and remodeling. It therefore plays an important role in many physiological, reactive, and pathological processes [1]. Angiogenesis requires specific morphogenetic responses of the two principal vascular cell types, namely endothelial cells and mural cells (pericytes and vascular smooth muscle cells), which need to migrate, proliferate, polarize and form a lumen, and deposit a basement membrane. Each sprout is led by a specialized endothelial tip-cell, which responds to attractive and repulsive cues presented by the surrounding tissue. The major known attractive cue, vascular endothelial growth factor–A (VEGF-A), binds to VEGF receptors (primarily VEGFR2) on tip-cells to promote the formation and extension of filopodia in the direction of a gradient or immobilized source of VEGF-A. The formation of the proper number of tip-cells is regulated by delta-like ligand 4/(dll4)/Notch receptor signaling, which forms a lateral inhibitory circuitry, whereby VEGF triggers expression of dll4, which in turn inhibits the VEGF responsiveness, and hence the induction of the tip-cell phenotype in neighboring endothelial cells [2], [3], [4], [5], [6].

Apart from endothelial and mural cells, various other cell types in the surrounding tissue regulate the angiogenic process. For example, astrocytes play a pivotal role during developmental angiogenesis in the retina. Astrocytes distribute ahead of the growing vascular front, forming a scaffold at the retinal surface onto which the primitive vascular network is organized [7], [8]. Retinal astrocytes also release VEGF-A in response to hypoxia in the avascular part of the retina [8], [9]. Astroglial cells related to the retinal astrocytes fulfill similar functions in other parts of the central nervous system (CNS), like the radial glial cells that guide angiogenic sprouts in the developing hindbrain [10] and in the deeper parts of the retina. Outside the CNS, other cell types constitute the preferential sources of VEGF-A and provide scaffolds or matrices onto which the endothelial cells migrate and form vascular networks. These cells may be epithelial and highly organ-specific, such as the podocytes of the kidney glomerulus [11], or mesenchymal and widely distributed (albeit not universal), such as fibroblasts.

In contrast to the abovementioned cell types, tissue macrophages constitute a regulatory cell type that appears to be universally associated with angiogenesis during developmental and pathological angiogenesis. Macrophages may hence play a general role in these processes, a role that, however, remains ill defined. In general, macrophages appear to be pro-angiogenic, and it has been proposed that they mediate the angiogenic effects of placenta growth factor (PlGF) and macrophage colony-stimulating factor/colony stimulating factor-1 (M-CSF/CSF-1) in both therapeutical and pathological conditions [12], [13], [14]. Moreover, macrophages or macrophage-like cells have been proposed to promote angiogenesis in tumors and in situations of ectopic VEGF expression [15], [16].

Microglia is the term often used for tissue macrophages residing in the CNS. Microglia are a heterogeneous population of bone marrow-derived monocytes/macrophages [17], [18], [19], [20] that invades the brain during early embryonic development [21]. In the retina, microglial cells are in close contact with developing blood vessels, and the presence of microglia has been correlated with both developmental [14], [22], [23] and pathological angiogenesis [14], [22], [24], [25]. Microglia are lost in conjunction with retinopathies associated with blood vessel loss, and chlodronate-mediated depletion of microglia coincide with reduced retinal vessel formation during development that can be restored by retinal injections of microglia [22]. Kubota and co-workers found that macrophages constitute the M-CSF effector cells, which in turn promote angiogenic responses both in tumors and during developmental retinal angiogenesis [14]. Using CSF-1 deficient *csf-1* ^op/op^ mice, they showed that absence of microglia in the postnatal mouse retina correlated with the formation of a sparser than normal retinal vessel network. The recent study by Fantin and co-workers [23] provided a spatial correlation between tip-cells of angiogenic sprouts and the occurrence of microglia at several locations in the developing mouse CNS and during intersomitic vessel formation in the zebrafish embryo [23]. Their study also showed that absence of microglia correlated with fewer points of contact between neighboring tip-cells, and that the microglial effect appears additive to the effect of VEGF. Based on these observations, Fantin and co-workers proposed that microglia provide scaffolds for sprout fusion [23].

In the present study we confirm that microglia occur preferentially at sites of sprout anastomosis formation during retinal developmental angiogenesis, and that vascular network formation is impaired in the genetic absence of microglia. By using an *ex vivo*/*in vitro* model of angiogenesis, we show that the angiogenic effect can be obtained by ectopically added microglia alone and, as in the *in vivo* situation, that the microglial effect appears not to be mediated by VEGF-A. Importantly, we found that the microglial effect is mediated through a mechanism that involves a soluble product(s) of microglia cells, rather than direct cell contact. Conversely, the growing vessels appear to secrete factors to attract microglia, which may contribute to the localization of microglia at the vascular front. Our findings, revealing a two-way communication between microglia and vessels that depends on soluble factors, advance the understanding of how microglia promote vascular network formation.

## Results

### Microglial cells associate with vessels sprout anastomoses during developmental angiogenesis in the mouse retina

**Figure 1:**
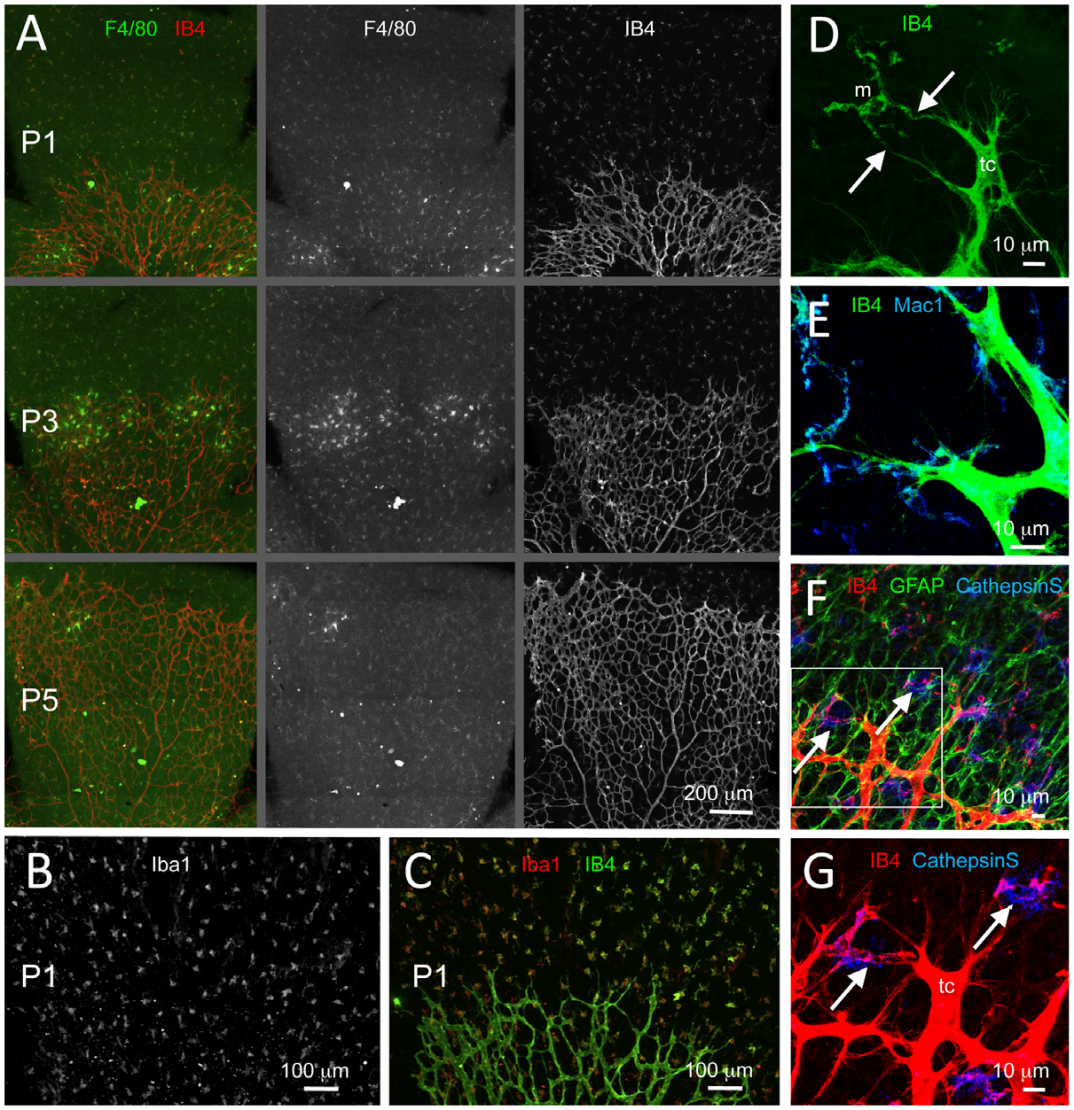
Spatial relationship between microglia and endothelial cells during retinal development in the mouse. (**A**) Fluorescence microscopy of IB4 and F4/80 double-stained flat-mounted retinas from newborn mice at postnatal days (P) 1 (P0; day of birth), 3 (P3) and 5 (P5). Note the presence of dispersed IB4 and F4/80 double positive microglial cells ahead of the developing retinal vascular plexus. (**B,C**) P1 retina double-stained for IB4 and the microglial cell marker Iba1; (**B**) shows Iba1 staining only, whereas (**C**) shows double staining. (**D–G**) Identification of microglia by marker expression and their spatial relationship with tip-cells. Note in (D) how tip-cells (tc) protrude their filopodia towards microglial cells (m). Arrows in (D) point at contacts made between tip-cell filopodia and microglial cytoplasmic protrusions. In (E–G), microglial cells are stained using antibodies against Mac1 and mRNA in situ hybridization for CathepsinS (NBT/BCIP signal is visualized using 633 nm laser reflection). In (F) co-staining of astrocytes for glial fibrillary acidic protein (GFAP) shows the difference in density and distribution between astrocytes and microglia, the latter appearing as solitary cells located within a dense and continuous layer of astrocytes organized in a network structure. In (F,G), which represent the same area at different magnifications and with and without GFAP staining, the left arrow points at a microglial cell situated at a point of contact between two neighboring tip-cells. The right arrow points at a tip-cell extending filopodia towards a microglial cell situated in front of the vascular plexus.

Microglial cells, like endothelial cells, are strongly labeled by *Griffonia simplicifolia* isolectin-B4 (IB4) (Fig 1A,D). Their characteristic morphology makes them readily distinguishable from the endothelial cells in IB4-labeled specimen, where whole cells may be observed, such as in whole-mount preparations of the mouse retina and in thick tissue sections of the CNS (Fig 1A,D and data not shown). We also used F4/80, Iba1, Mac-1 and cathepsin S to distinguish microglia cells in close contact with endothelial cells (Fig 1A–C,E–G and data not shown).

Microglia invade the mouse retina prior to vascularization (Fig 1A–C). During the first postnatal week of development, microglial cells are distributed as singular cells in a regular density at the retinal surface ahead of the growing vascular plexus (Fig 1A–C). Co-staining of microglia and endothelial cells demonstrate that microglia cells are commonly associated with endothelial tip-cells, i.e. the cells that lead angiogenic sprouts (Fig 1D). Tip-cells have a characteristic morphology, including long filopodial protrusions (exemplified in Fig 1D,G), making them distinguishable from the lumen-forming stalk-cells. In particular, microglial cells were commonly found to be present at sites were two tip-cells are contacting each other through their filopodia at sites of prospective sprout anastomosis (examples in Fig 1F,G, left arrows). This relationship was also observed in other parts of the developing mouse CNS (data not shown).

### Absence of microglia correlates with the formation of a sparser network of vessels in the developing retina

The presence of microglia at sites of contact between neighboring endothelial tip-cells suggested that microglial cells play an active role in promoting angiogenic sprout anastomosis formation. By determining the density of microglial cells in front of the forming retinal vascular plexus on F4/80 and Iba1 labeled flat-mounted retinas and comparing it with the density of vessel branch points immediately behind the sprouting front using IB4-co-labeled specimen in P1–P3 retinas, we found the ratio between the two to be close to 1∶2 (data not shown), which would be the expected ratio if every microglia cells gets engaged in a vascular anastomosis event in early postnatal retinal development.

**Figure 2:**
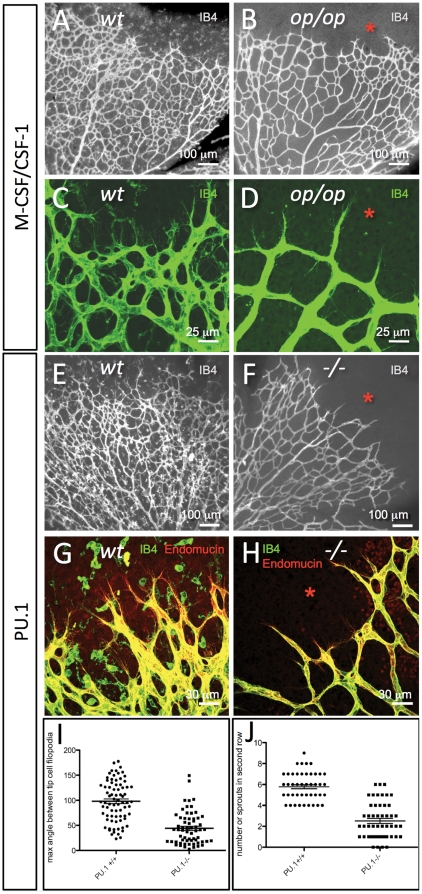
Absence of microglial cells correlates with morphological changes in tip-cells and in the developing retinal vascular plexus. (**A–D**) Retinal vascular plexus morphology as depicted by IB4-staining in P6 mice of wildtype (A,C) or *csf-1^op/op^* genotype (B,D). Note the complete absence of IB4-stained microglial cells in the retina ahead of the vascular plexus (asterisks), and the difference in vascular density. Note also that the tip-cells and their filopodia in the *csf-1^op/op^* retinas (D) are more uniformly radial in their orientation than the corresponding tip-cells of the control (C). (**E–H**) Retinal vascular plexus morphology as depicted by IB4/Endomucin staining in P6 mice of wildtype (E,G) or *Pu.1^-/-^* genotype (F,H). Note the absence of microglial cells (asterisks), the sparser developing vascular plexus and a preferential radial orientation of tip-cell filopodia in the mutants (F,H). (**I, J**) The maximum angle between the filopodia protruding from a single tip cell is reduced in *Pu.1^-/-^* mice (I, n = 76, p<0.0001), and less tip-cells and filopodia were observed in the second row of branches, i.e. at a more central location in the retina of *Pu.1^-/-^* mice (J, n = 50, p<0.0001), suggesting that microglia promotes further angiogenic sprouting at this location.

To explore this idea further, we studied retinal vascular formation in M-CSF/CSF-1 deficient mice. Osteopetrotic *csf-1* ^op/op^ mice are homozygous for an inactivating mutation in the *csf-1* gene, and hence lack the CSF-1 protein [26]. As a consequence, *csf-1* ^op/op^ mice lack microglial cells in the retina during the first week of postnatal development [14] (Fig 2B, D). We found that *csf-1* ^op/op^ mice developed a significantly sparser vessel network compared to controls during this developmental period (Fig 2A–D). Also, the sprouts in *csf-1* ^op/op^ mice were more radially oriented than sprouts in controls (compare Fig 2D with C).

Effects similar to those in *csf-1* ^op/op^ mice were observed in PU.1 knockout mice [27] (Fig 2E–H). PU.1 is an Ets family transcription factor that regulates hematopoietic cells' responsiveness to, and expression of receptors for, M-CSF, GM-CSF and G-CSF [28]. PU.1 null mice lack monocyte/macrophage cells, and also B-cells. As a consequence, they lack the monocyte-derived retinal microglial cells [23] (Fig 2F,H). PU.1 null mice developed a sparser retinal vessel network than controls, and, like the *csf-1* ^op/op^ mice, their endothelial tip-cells were more radially oriented than in controls (Fig 2C,D,G,H). In search for a mechanistic basis for this change in tip-cell orientation, we studied the orientation of the tip-cell filopodia. Consistent with previously reported observations [14], [23], the number of tip-cells at the plexus front was not significantly different in the microglial deficient mutants compared to controls (data not shown). However, the brush of filopodia extending from each tip-cell in PU.1 null mice had on average a significantly more acute angle (≈50°) compared to controls (≈100°) as assessed by the angle between the two most lateral filopodia in the brush (Fig 2I). Also, the number of filopodia-bearing sprouts present in the vascular plexus behind the front was significantly smaller in PU.1 knockouts compared to controls, suggesting that microglia may promote (secondary) sprouting in the retinal plexus (Fig 2J).

### Microglia stimulate vessel sprouting and branching *in vitro*

Our *in vivo* observations establish a correlation between the presence of microglia and the formation of vessel anastomoses and secondary angiogenic sprouting during developmental angiogenesis in the mouse retina. To test if microglial cells are sufficient to induce vessel branching when added to an angiogenic situation, we modified and adapted the rat aortic ring culture system described earlier [29] to the mouse. This assay reproduces several aspects of angiogenesis *in vivo*, with the important difference that it disconnects the angiogenic sprouting process from potential systemic confounders such as blood flow, blood pressure and hemostatic regulation [29].

**Figure 3:**
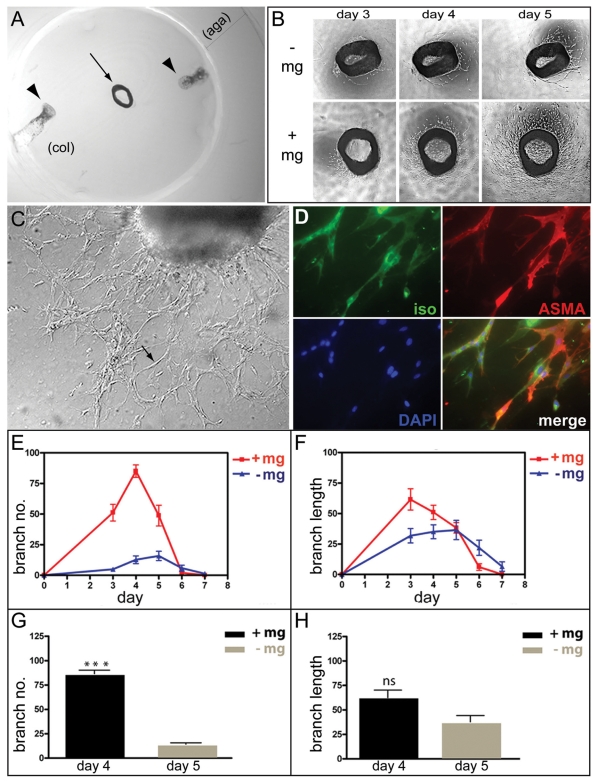
Microglia stimulate angiogenesis. (**A**) An aortic ring explant (arrow) embedded between two disks of collagen type Ι (col) and surrounded by a cylinder of agarose type VII (aga). Microglia cells in suspension were injected between the discs at two locations (indicated by arrowheads). (**B**) Phase-contrast images of aortic rings explants from one mouse cultured in the absence (-mg) or in the presence (+mg) of microglia. The images show the same aortic ring explants after three, four and five days in culture. (**C**) An inverted microscope image showing an aortic ring culture with neovessels at day 4 (arrow points to a vessel). (**D**) Whole mount immuno-staining of an aortic ring co-cultured with microglia. The aortic ring explant was stained with IB4 (green) for endothelial cells, mouse monoclonal anti-α-smooth muscle actin antibody (red) for pericytes, and DAPI for nucleic acids (blue), and their merged image is shown. (**E,F**) Numbers and lengths of the vascular branches in 24 aortic ring explants (from four mice) cultured with or without microglia were determined each day for 7 days. The diagrams indicate the mean relative values (the ratio between the particular value and the highest value in the specific series) for number (E) and length (F) of the vascular branches of four independent series of experiments. (**G** and **H**) Mean relative values obtained on the day of maximum angiogenic response (day four for cultures in the presence of microglia and at day five for cultures in the absence of microglia) in terms of number (G) and length (H) of the vessels in the four independent experiments above. Bars indicate standard errors of the means. The angiogenic response of aortic rings in terms of branch number was significantly increased in the presence of microglia (***p<0.001), whereas the difference in response in terms of branch length was not statistically significant (ns).

Mouse aortic rings were cultured in collagen gels with serum, but otherwise in the absence of added growth factors. Microglial cells were deposited locally in the gel (Fig 3A). Four series of mouse aortic ring explants prepared from four mice at different occasions, were incubated in the presence or absence of microglia cells (12 rings for each situation; see Methods and Fig. 3). In the presence of microglia the aortic rings began to produce sprouts after 2 days in culture compared to after 3 days in the absence of added microglia cells. The incubations were continued for one week and the number of branches and length of the neovessels were determined daily by microscopy (Fig 3B,C). Between day 3 and day 5, aortic rings co-cultured with microglial cells displayed a several-fold increase in the number of branches as compared with rings cultured in the absence of microglia. The peak responses with and without added microglia occurred on day 4 and 5, respectively, i.e. with the same time difference (one day) as for the induction of sprouts (Fig 3B,E). A possible slight stimulatory effect on length of the angiogenic sprouts was also observed (Fig 3F). Statistical analysis of the peak responses showed that the peak vessel branch number was considerably higher in the presence than in the absence of microglia (p<0.0001), whereas the difference in response in terms of peak branch length was not statistically significant (Fig 3G, H). In these experiments, 200,000 microglia cells were added to each aortic ring culture. In dose-response experiments, the effect on vessel sprouting increased with increasing number of added microglia cells up to 200,000.

To confirm that the vessel sprouts (shown in high magnification in Fig 3C) represented endothelial cells together with associated pericytes, aortic rings cultured with or without microglial cells were whole mount fixed and stained for endothelial cells (IB4), pericytes (α-smooth muscle actin) and nuclei (DAPI). These analyses demonstrated that the branches were composed of both endothelial cells and pericytes at similar proportions whether or not microglia were added (Fig 3D and not shown). Taken together, these results suggest that microglial cells have a stimulatory effect on angiogenic sprout formation and branching *in vitro* in the mouse aortic ring model.

### Angiogenic effect of microglia is cell type-specific

**Figure 4:**
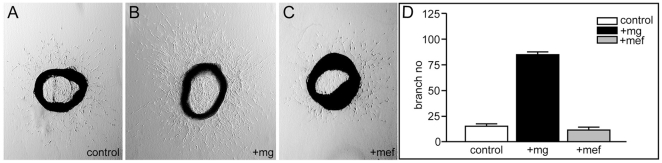
The angiogenic effect of microglial cells is cell type-specific. (**A–C**) Mouse aortic ring explants were co-cultured in triplicate with microglial cells (B), mouse embryo fibroblasts (C) or medium only (A). Shown are representative phase contrast microscopy images of the aortic ring cultures at day five. (**D**) The number of vascular branches generated by the rings was determined and normalized for each series of four independent experiments with four animals. The mean relative number of branches for the 12 rings per condition is presented by the bar graph with standard errors of the means.

We next asked if the angiogenic effect of microglia in the aortic ring model was cell type-specific or could be mimicked by other mesenchymal cells. We therefore compared the effect of microglial cells on branching of aortic ring vessels with that of mouse embryonic fibroblasts (MEF). Thirty-six aortic ring explants from 4 different animals were prepared, of which 12 were co-cultured with microglia, 12 with MEF and 12 cultured without added cells. The number of branches formed by each aortic ring was determined on day 5 of culturing (Fig 4A–C). Consistent with previous experiments, statistical analysis of the result showed that microglia induced a 5-10 fold increase in branch number (p<0.0001) (Fig 4D). In contrast, the addition of MEF did not increase the number of branches, but slightly decreased branching in comparison with aortic rings with no cells added. A possible explanation of this latter observation would be that the MEF competed for and consumed growth stimulatory factors in the medium. These results support the notion that the stimulatory effect of microglia on vessel branching is specific for this cell type.

### Direct cell-to-cell contact is not essential for the angiogenic stimulatory effect of microglia cells

**Figure 5:**
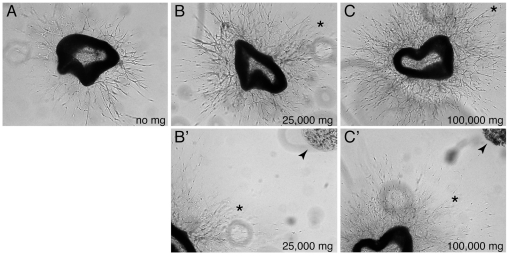
The angiogenic stimulatory effect of microglia cells does not depend on direct cell-to-cell contact. (**A–C**) Dose-response of microglial cells immobilized in collagen gel in aortic ring cultures. Phase contrast images were obtained at day four for cultures with no added microglia (A), with 25000 microglia cells (B) and with 100000 microglia cells (C). B and C show the central part of the cultures with the aortic ring surrounded by neovessels, while B' and C' also include the embedded microglia cells (arrowheads). Corresponding positions in the respective images are marked with asterisks.

In our aortic ring cultures, the applied microglial cells spread from their site of injection to finally infiltrate the endothelial network. An important question is therefore whether microglia stimulate vessel branching through direct contacts with the endothelial network, or indirectly via soluble factors, or both. To address this question we took advantage of the fact that the microglial cells migrated with a much-reduced velocity when embedded in collagen gel upon injection. When comparing aortic rings cultured with or without such embedded microglia, it was apparent that the microglia induced sprouting long before the cells had made physical contact with the growing vessel network (Fig 5). Microscopic analysis demonstrated a dose-dependent stimulatory angiogenic effect of microglial cells on vessel branching (Fig 5B–C). From these experiments we conclude that microglial cells release a soluble factor(s) that stimulates sprouting from the aortic rings.

### Aortic ring explants induce secretion of stimulatory angiogenic factors from microglial cells and directed migration of the cells towards the explants

**Figure 6:**
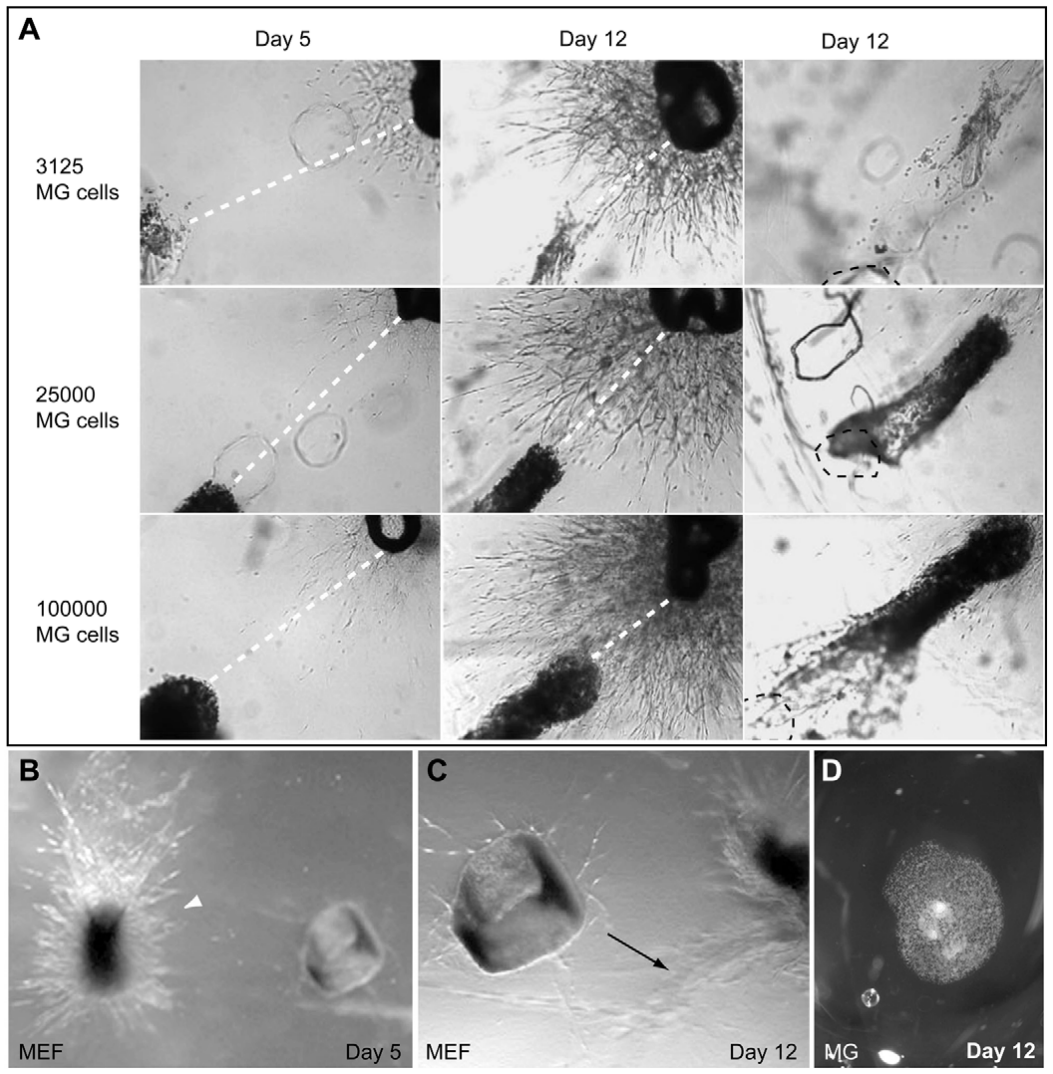
Aortic ring explants induce migration of microglial cells towards the explants. (**A**) Aortic ring explants were co-cultured for 12 days with different numbers of microglial cells embedded in collagen and were monitored by phase contrast microscopy. The distance between the aortic ring and the aorta-proximal border of the bulk of microglial cells was measured daily. Images from days 5 and 12 are shown, and stippled white lines indicate the measured decreasing distances. The images in the right panel show the original site of application of the microglial cells, which is marked by a stippled black line. (**B,C**) Aortic ring explants were co-cultured with mouse embryonic fibroblasts (MEFs) under conditions similar to those in (A). The MEFs exhibited radial growth (arrowhead in B) and, when approaching the vessels, turned away from the aorta ring (arrow in C). (**D**) Collagen-embedded microglial cells without the presence of an aortic ring spread radially.

We consistently observed that microglia exhibited directed migration towards the aortic rings, which was independent of gel contraction. Such migration was also observed when microglial cells were suspended in a defined volume of collagen matrix prior to injection, which retarded their migration rate. The concerted movement of the cells in the gel could then be monitored over several days (Fig 6A). Aortic ring explants were co-cultured for 12 days with different numbers of microglial cells embedded in collagen, and the migration of the cells was monitored daily by phase contrast microscopy. A microglial cell dose-dependent formation of neovessels from the aortic rings was evident on day 3 when the microglia still remained at the application site. The microglia began to migrate towards the aortic ring on approximately day 4 of culturing. Figure 6A illustrates the position of microglia at day 5 and 12 for cultures containing 3,125, 25,000 and 100,000 microglial cells. The distances between the front of the migrating microglia and the aortic ring decreased by approximately 1mm from day 5 to day 12, yielding a migration rate corresponding to about 140 µm per day. Parallel experiments in which MEFs replaced the microglia showed a strikingly different pattern of cell migration (Fig 6B). In contrast to the oriented migration exhibited by microglia, the MEFs spread radially in all directions from the site of injection, as did microglia in the absence of an aortic ring (Fig 6D). When approaching the aortic ring, the MEFs changed direction and turned away from the vessels (Fig 6C). This supports the notion that the induced migration of microglial cells towards the endothelium aortic ring explant is cell type-specific.

**Figure 7:**
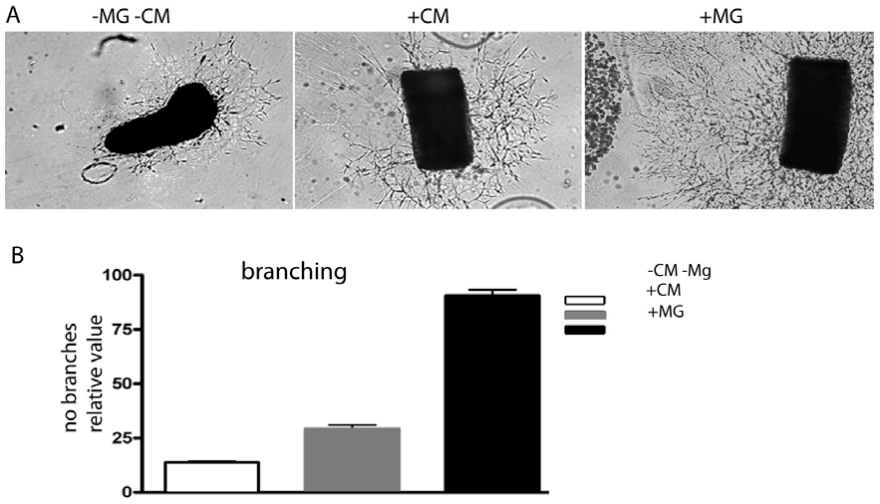
The angiogenic response of aortic rings in culture is significantly higher in the presence of activated microglial cells compared with in conditioned medium only. (**A**) Phase-contrast images of cultured mouse aortic ring explants with standard culture medium (left), microglia conditioned medium (middle) and with microglia (right). The images show aortic rings at day five of culturing. (**B**) Diagram showing the mean number of vascular branches in 12 aortic rings for each experimental condition. Conditioned medium was obtained from the same number of microglia as was applied in the co-culture experiment. Bars indicate standard errors of the means. The angiogenic response of aortic rings cultured in the presence of microglia cells was significantly higher than that of aorta rings grown in conditioned medium (p<0.001).

These results indicated that microglial cells secrete a soluble factor(s) into the aortic ring culture medium that stimulated vessel branching in the explants. The results also suggest that the aortic rings affect microglial cell migration in the collagen gel. To address if aortic rings also influenced the release of angiogenesis stimulatory factor(s) from microglial cells, the effects of cell-free microglia conditioned and control medium were compared with embedded microglia in the aortic ring model. Conditioned medium was obtained from microglial cell cultures incubated in parallel with the aortic ring cultures in the same standard medium and with a similar number of cells. When comparing branch numbers on day 5, large differences in vessel sprouting were observed (p<0.001, n = 9) between cultures with embedded microglial cells and cultures supplemented with microglial cell conditioned medium (Fig 7). Furthermore, a smaller but significant difference in vessel sprouting (p<0.05, n = 9) was observed when comparing microglial cell conditioned medium with control medium (Fig 7). These results suggest that microglial cells secrete a soluble factor(s) with a positive angiogenic effect on the aortic ring explants and that the secretory activity of the microglial cells is stimulated by the presence of aortic ring explants in the cultures.

### The microglial cell-derived angiogenic factor(s) is distinct from VEGF-A

**Figure 8:**
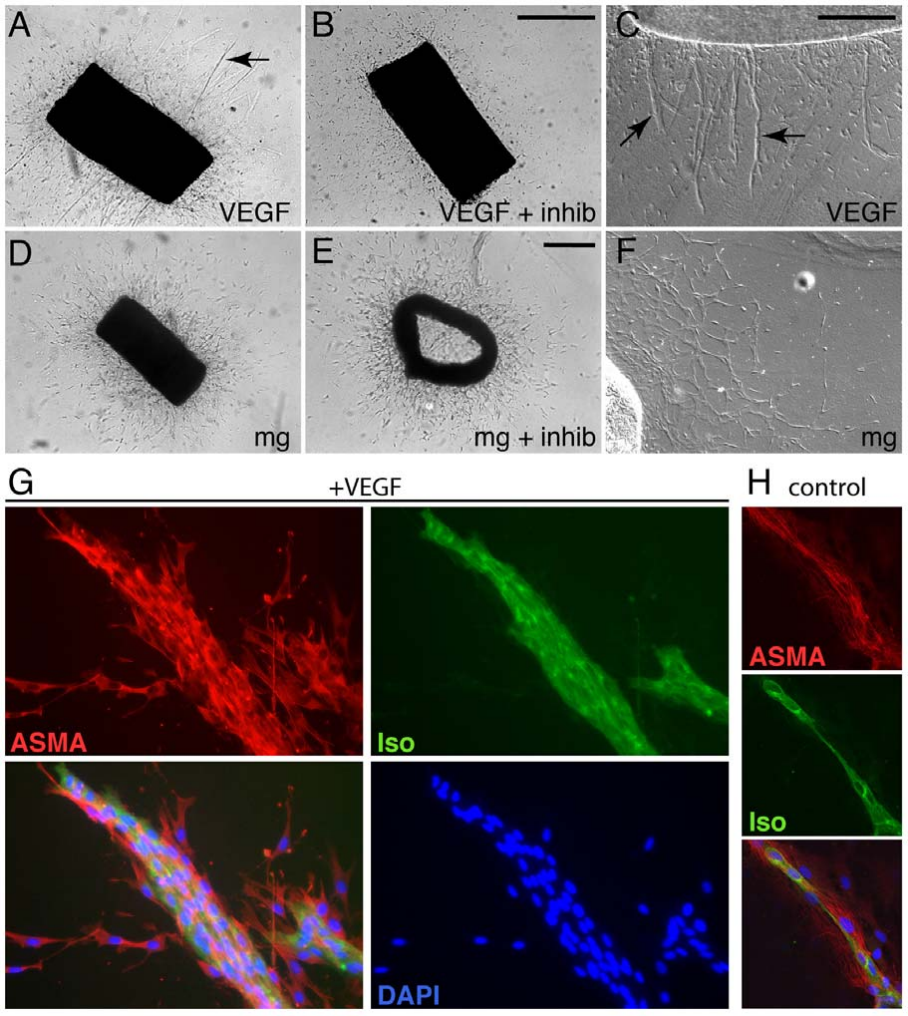
The angiogenic effect of microglia is independent of VEGF-A. (**A–F**) Aorta ring explants stimulated with VEGF-A (A and C), VEGF-A plus soluble VEGFR1 (B), microglia (D and F) and microglia plus soluble VEGFR1 (E) are shown by light microscopy. C and F are higher magnification images using differential interference contrast microscopy. Arrows in A and C point to robust microvessels induced by VEGF-A. (**G–H**) Staining of sprouts with IB4 (green, for endothelial cells) alpha-smooth muscle actin antibodies (ASMA, red, for mural cells) and DAPI (blue, nuclei) alone or in combination. A typical sprout morphology in the presence of VEGF-A is shown in (G) and the corresponding morphology in the presence of microglia is shown in (H).

The most pivotal regulator of blood vessel formation *in vivo* is VEGF-A, which is produced by many cell types, including myeloid cells [30]. To investigate if the angiogenic response of the aortic ring explants induced by microglial cells was mediated by VEGF, we added VEGF-A (*mouse VEGF 164,* R&D) at concentrations ranging between 5 to 40 ng/ml plus or minus a soluble VEGF receptor-1 (VEGFR1) chimeric molecule (*VEGFR1/Flt-1/Fc Chimera*, R&D, at 400 ng/ml) that acts as an extracellular trap for VEGF-A, VEGF-B and PlGF. Added VEGF-A induced formation of vessels from aortic rings in a dose-dependent manner (Fig 8A and data not shown). This effect was phenotypically different from the effect of added microglia in that the vascular sprouts induced by VEGF-A were thicker and contained more endothelial cells (compare Fig 8A with Fig 8D, Fig 8C with 8F, and Fig 8G with Fig 3D). As expected, the VEGF-A effect was inhibited by the simultaneous addition of 400 ng/ml of soluble VEGFR1 (Fig 8B). We next compared the stimulatory effect of microglia on branching of aortic ring vessels in the presence of soluble VEGFR1 (Fig 8E). Fifty-two aortic ring explants from 4 animals were prepared of which 38 were co-cultured with 200,000 microglial cells. Soluble VEGFR1 was added to 19 cultures. At least one explant in each series received VEGF-A and one received VEGF-A plus soluble VEGFR1. These experiments demonstrated that whereas the effect of VEGF-A was invariably inhibited by soluble VEGFR1, the microglia-induced sprouts were unaffected (Fig 8E). This suggests that the angiogenic factor(s) released from microglial cells is distinct from VEGF-A (and VEGF-B and PlGF). Our findings are consistent with previous reports showing that microglial cells in the developing mouse CNS do not express detectable levels of VEGF-A mRNA [8]. In other experiments we tested the possible effect of a VEGFR1 neutralizing antibody. The results from these studies failed to reveal any evidence for a role of VEGFR1 in microglia or VEGF-A induced vessel sprouting from aortic rings (data not shown).

## Discussion

In this study, we used the developing mouse retina and the aortic ring model to address the role of microglial cells in angiogenesis. The retina is an organ where too many or to few vessels are associated with pathology. The retina is also subject to pharmacological application of anti-VEGF therapy, which is used to counteract the edema that compromises vision in age-dependent macula degeneration [31], [32]. This clinical relevance combined with the many advantages of the retina for experimental studies of angiogenesis makes it an ideal location to study the effect of angiogenic modulators. Accordingly, the retina is also a suitable location to study the influence on angiogenesis of non-vascular cell types such as microglial cells. The aortic ring model reproduces angiogenic sprouting in culture in three-dimensional biomatrix gels [33]. The vessel outgrowths produced by aortic rings consist of endothelial cells in interaction with mural cells as well as other types of mesenchymal cells, such as fibroblasts and macrophages [34]. Because the aortic ring model is intermediate between simpler *in vitro* models of angiogenesis and complex *in vivo* models, the aortic ring model has become attractive as a reproducible and relatively high-throughput assay for the study of angiogenesis. Hence it has been broadly used for the study of basic mechanisms of angiogenesis, and to test the effects on angiogenesis of diverse components, such as growth factors and cytokines, immune regulatory molecules, proangiogenic or antiangiogenic compounds, protease inhibitors, extracellular matrix components and their receptors, and different cell types (reviewed in [34]).

Our observations *in vivo* suggest that microglial cells exert a stimulatory effect on angiogenesis. These observations are consistent with those of Fantin and colleagues [23], that tissue macrophages/microglia are associated with angiogenesis in different developmental organs. However, the *in vivo* studies fail to show if the effect of microglial cells on angiogenesis is direct or indirect. Our *in vitro* findings demonstrate that microglial cells directly promote vessel sprouting in the aortic ring model, and are consistent with a general stimulatory role of microglia on angiogenesis. Our observations in the developing retina also suggest that microglia affect the number and direction of filopodial extensions from tip-cells at the vascular front and within the forming retinal vessel plexus. In the presence of microglia, endothelial tip-cell filopodia protruded both in a forward (radial) orientation - towards the avascular retinal periphery - and sideways towards microglial cells and other tip-cells, resulting in filopodial brushes with obtuse angle. In the absence of microglia the tip-cell filopodial protrusions were mainly oriented forward. These observations are consistent with a model in which microglial cells exert their angiogenic effect by promoting the protrusion and/or stabilization of endothelial tip-cell filopodia.

Although the VEGF/Notch signaling loop likely constitutes the basic signaling machinery that ensures formation of the nascent vascular network, it is clear that there must be other modulators of the system. Fantin and coworkers reported that the microglia-derived angiogenic activity acts parallel to VEGF-A, since the effects of microglia and VEGF-A appeared additive when studied in genetic mouse models [23]. In the aortic ring model, addition of microglia promoted formation of a fine network of branches with one to two cells at the branch circumference. In contrast, addition of VEGF-A promoted formation of thicker branches with multiple cells at the branch circumference. Importantly, while addition of the VEGF inhibitor could reverse the effect of VEGF-A, it had no profound effect on microglial-induced vessel branching. Thus, our findings in the aortic ring model are consistent with the *in vivo* observations reported herein and previously.

In this context it is of interest that resident macrophages present in the aortic ring have been reported to play a permissive role in the angiogenic response from the ring explants; macrophage depletion inhibits angiogenic responses in the rings [35]. The same group also reported that a subset of immature immune cells grown out from aortic ring cultures stimulate angiogenesis in freshly cut rings [36]. However, in contrast to the microglial cells used in this study, those cells produced significant quantities of VEGF-A, suggesting that different sources and phenotypes of leukocytes may affect angiogenic responses via different mechanisms.

A attractive explanation for the *in vivo* observations that microglia localize near sites of endothelial tip-cells, would be that microglia act as guideposts for anastomosis formation during development of the vascular network that initially covers the retina. This model would imply (although not necessarily demand) direct contact between microglial cells and endothelial tip-cells as a critical step in the anastomosis process. Intriguingly, however, using the aortic ring model, we found that microglia induced sprouting in the absence of a direct contact with the vessels. Moreover, conditioned medium from microglia could partly mimic the effect of ectopically added microglia. Thus, at least some of the effect of microglia on angiogenesis seems to rely on a secreted factor(s). Together, our *in vivo* and *in vitro* observations would suggest that microglia provide a signal(s) for filopodial protrusion from endothelial tip-cells, a signal that competes with the VEGF-A produced by retinal astrocytes. Inflammation is also known to activate endothelial cells and promote vessel branching. However, when assaying a panel of inflammatory cytokines, we failed to detect any up-regulation of such cytokines in media form aortic rings cultured with microglia compared to media from control aorta ring cultures (SR and CB, unpublished), and further studies will be required to identify and characterize the microglia-derived signal(s).

Our results using aortic ring explants further suggest that the close association of microglia with the vascular front and endothelial tip cells observed *in vivo* may depend on vessel-derived secretion of microglial attractant(s). Furthermore, the angiogenic effect of microglial cells was significantly stronger when they were exposed to aortic rings; plain microglial conditioned medium was angiogenic but the effect was weaker compared to the co-culture situation. Together, these observations suggest a two-way communication between the vasculature and the microglial cells, in which the vasculature attracts the microglial cells and promotes their release of angiogenic factor(s).

## Materials and Methods

### Animals

Animal housing and experiments were carried out in strict accordance with Swedish, US and UK legislation. Experiments performed were approved by the local animal ethics committees at Karolinska Institute (Committee North approvals N28/06, N73/07 and N33/10) and University of Gothenburg (approvals 279/04 and 277/09), respectively, prior to experimentation. Mutants were bred and genotyped as described for each mutant [26], [27].

### Retina preparation and stainings

Retinas were dissected and flat-mounted from mice of different genotypes and postnatal ages as described previously [8]. The techniques have been described before [8], including most reagents and procedures used to stain endothelial, cells, astrocytes and microglia, as well as the methods applied in order to visualize and count endothelial tip-cell filopodia. Additional antibodies used included rat anti-mouse F4/80 (Alexa Fluor 488 conjugated; CALTAG Laboratories, product code MF48020) diluted 1∶50 in PBS, 1% BSA, 0.2% Tween-20; rabbit anti Iba1 (Wako Chemicals, code no 019-19741) diluted 1∶200 in PBS, 1%BSA, 0.2% Tween-20 and rat-anti endomucin (clone V.7C7 Santa Cruz sc-65495) diluted 1∶50 in PBlec buffer as described [8]. Incubations were overnight at 4 degrees. Measurement of filopodia angles was performed using ImageJ v1.40g (http://rsb.ingo.nih.gov/ij/).

### Cells and culture conditions

Microglial cells: Mouse EOC 2 cell line was purchased from ATCC (catalog no. CRL-2467). EOC 2 is an immortalized microglial cell line derived from the brain of an apparently normal 10-day-old mouse [pub med: 8550814]. The microglial cells depend on growth factor colony stimulating factor 1 (CSF-1) for growth and do not constitutively express high levels of the major histocompatibility complex (MHC). The cells were cultured in Dulbecco's modified Eagle's medium with 4 mM L-glutamine modified by ATCC (catalog no. 30-2002) to contain 4.5 g/L glucose, 1.5 g/L sodium bicarbonate and 1.0 mM sodium pyruvate. The medium was supplemented with 10% fetal bovine serum, 20% LADMAC conditioned medium (CSF-1), penicillin (100 u/ml) and streptomycin (100 µg/ml). Adherent microglial cells were isolated by scraping and sub-cultured in new culture vessels after dilution 1∶2. Cultures were incubated at 37°C in 5% CO_2_ and the medium was changed every 2-3 days.

LADMAC cells**:** Mouse LADMAC cells were purchased from ATCC (catalog no. CRL-2420). LADMAC is a transformed cell line derived from mouse bone marrow cells highly enriched for macrophage progenitors after transfection with human cellular myc-homologous DNA sequences in the pBR325 plasmid (pR myc). LADMAC cells secrete the growth factor colony-stimulating factor 1 (CSF-1). CSF-1 is capable of supporting *in vitro* proliferation of mouse bone marrow macrophages. LADMAC cells were grown in Eagle's Essential medium with Earle's BSS and 2 mM L-glutamine (EMEM) modified by ATCC (catalog no. 30-2003) to contain 1.0 mM sodium pyruvate, 0.1 mM nonessential amino acids, 1.5 g/L sodium bicarbonate and supplemented with 10% fetal bovine serum, penicillin (100 u/ml) and streptomycin (100 µg/ml). Cultures were incubated at 37°C in an atmosphere containing 5% CO2. The sub-cultivation ratio was 1∶2 and the medium changed every 2–3 days.

MEF cells: Wildtype MEF cells were purchased from ATCC (catalog no. CRL-2752). The MEF cells were derived from embryonic fibroblast of *mouse musculus* with a 129Sv, C57Bl6 background. The MEF cells were grown in Dulbecco's modified Eagle's medium purchased from ATCC (Catalog no. 30-2002), supplemented with 10% fetal bovine, penicillin (100 u/ml) and streptomycin (100 µg/ml). Cultures were incubated at 37°C in 5% CO2 and sub-cultured at 80% confluence.

Conditioned medium: LADMAC conditioned medium was generated by culturing LADMAC cells in medium as described above until confluence. The medium was collected after 5 days, centrifuged, sterile filtered (200 nm filter), aliquoted and stored at -20°C.

### Reagents

Agarose type VII tested for cell culture and fetal bovine serum were purchased from Sigma-Aldrich. Collagen Ι (from rat tail, Gibco), Isolectin IB4, Biotin conjugated (molecular probes), Streptavidin, Alexa Flour conjugated 488 and 568 (molecular probes), were all obtained from Invitrogen. Purified rat anti-mouse *CD31* antibodies were purchased from BD Pharmingen.

### The aortic ring model

We modified and adapted the three dimensional aortic ring culture system from rat described by Nicosia [29] to the mouse. Thoracic aortas were dissected from isofluoran euthanized 9–12 week-old C57BL6 mice after perfusion for 5 minutes with cold CDMB 131 medium. The fibroadipose tissue was removed and the aortas were serially cross-sectioned into 1–2 mm rings. The aortic rings were embedded between two disks of collagen type Ι (1.5 mg/ml) with a diameter of 7 mm and surrounded by a ring of agarose type VII with a wall thickness of 3 mm. For co-culturing of aortic rings and cells, defined numbers of cells in suspension were injected in different positions around the aortic ring using a Hamilton syringe.

### Angiogenic response

The angiogenic response of the aorta rings was measured and documented in living culture, and at the end of the experiment the cultures were fixed in buffered 4% paraformaldehyde and processed for immunostaining. The number of branches and lengths of the microvessels were monitored on a daily basis, by examining the cultures under brightfield microscopy using non-phase inverted optics (Supplementary Fig S1). Microvascular sprouts were distinguished from fibroblasts based on their unique morphology (greater thickness and uniformly cohesive pattern of growth). Sprouts originating from the aorta ring are referred to as microvessels, and branches refer to sprouts originating from a microvessel or another branch. The following criteria were used when counting the number of branches [29]: (a) the dichotomous branching of one microvessel or branch to generate two new sprouts was scored as one new branch, and (b) each loop was counted as two branches, since such loops usually form by anastomosis between two converging sprouts. An example of branch count is presented in Supplementary Fig S1. Microvessel lengths were estimated by measuring the distance from the aorta ring to the tip of the microvessel (ten vessels per culture and at least two from each quarter sector of the ring were measured). The mean length of these ten branches was taken as the microvessel length of the aorta ring culture.

### Statistical analysis

Graph Pad Prism 5 software was used for statistical analysis. For three-group comparison, the analysis of variance (Anova) test was used. Paired experiments were compared using 1-tailed student's t-test.

## Supporting Information

Figure S1 **Estimation of branch number and microvessel length.**
(TIF)
