# Supplementary figures and images for: A Two-Way Communication between Microglial Cells and Angiogenic Sprouts Regulates Angiogenesis in Aortic Ring Cultures
Source: PLoS One. 2011 Jan 10;6(1):e15846. doi: 10.1371/journal.pone.0015846 (PMC3018482; doi:10.1371/journal.pone.0015846)

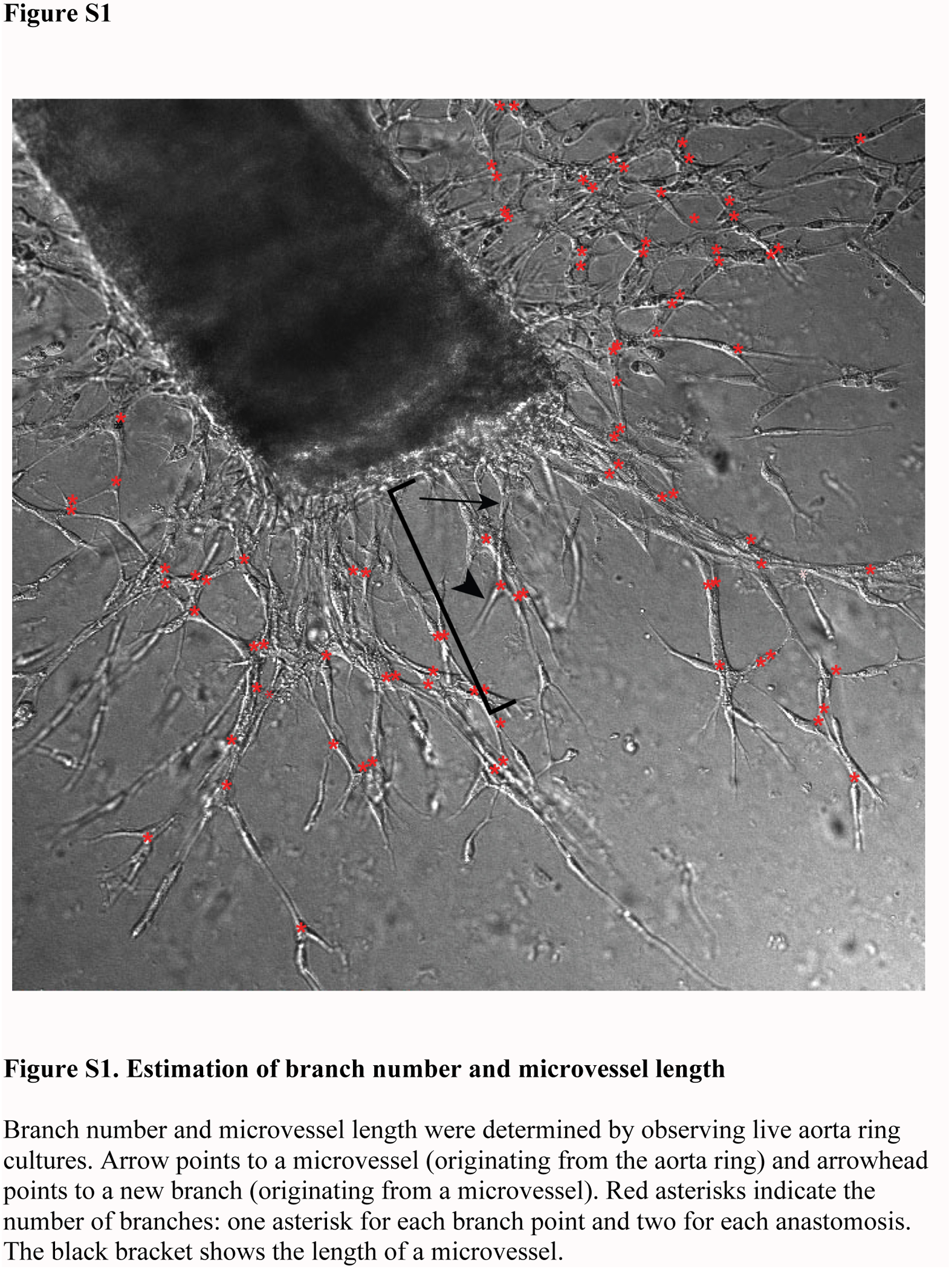

Supplement: Figure S1 — Estimation of branch number and microvessel length. (TIF) [file pone.0015846.s001.tif]
